# Investigation and Genome-Wide Association Analysis of Fusarium Seedling Blight Resistance in Chinese Elite Wheat Lines

**DOI:** 10.3389/fpls.2021.777494

**Published:** 2021-11-17

**Authors:** Yike Liu, Guang Zhu, Zhangwang Zhu, Lin Chen, Hongli Niu, Weijie He, Hanwen Tong, Jinghan Song, Yuqing Zhang, Dongfang Ma, Chunbao Gao

**Affiliations:** ^1^Hubei Key Laboratory of Food Crop Germplasm and Genetic Improvement, Food Crops Institute, Hubei Engineering and Technology Research, Hubei Academy of Agricultural Sciences, Wuhan, China; ^2^Center of Wheat, Wheat Disease Biology Research Station for Central China, Wuhan, China; ^3^Engineering Research Center of Ecology and Agricultural Use of Wetland, Ministry of Education, Yangtze University, Jingzhou, China; ^4^Hubei Collaborative Innovation Center for Grain Industry, College of Agriculture, Yangtze University, Jingzhou, China

**Keywords:** common wheat, Fusarium seedling blight, Fusarium head blight, GWAS, QTL

## Abstract

Fusarium seedling blight (FSB) is an important disease of wheat occurring as part of the Fusarium disease complex consisting also of Fusarium head blight (FHB). 240 Chinese elite cultivars and lines were evaluated in greenhouse experiments for FSB resistance and genotyped using the wheat 90 K single nucleotide polymorphism arrays. Among them, 23 accessions had an average lesion length of less than 0.6 cm, exhibiting potential for breeding for FSB resistance in wheat. Jingfumai 1 and Yangmai 11 had a relatively high resistance to both FSB and FHB simultaneously. Six relatively stable quantitative trait loci (QTLs) were detected on chromosome arms 1DL, 3AS, 3BL, 6BL, 7AL, and Un using the mixed linear model approach, interpreting 4.83–7.53% of phenotypic variation. There was a negative correlation between the average FSB lesion length and the BLUE FHB index with a low coefficient, and resistance to both diseases appeared to be conferred by different QTLs across the same population. Four KASP markers were detected on 1DL, 3AS, 3BL, and 6BL in QTLs to facilitate marker-assisted selection. Combined with transcriptome data analysis, eight defense-related genes were considered as candidates for mapping QTLs. The resistant elite germplasm, mapped QTLs, and KASP markers developed in this study are useful resources for enhancing Fusarium seedling blight in wheat breeding.

## Introduction

Fusarium seedling blight (FSB) and Fusarium head blight (FHB), primarily caused by Fusarium pathogens, refer to are economically devastating diseases in wheat (*Triticum aestivum L*.) as well as other small grain cereals across the world (Bai and Shaner, 2004; Li X. et al., 2010; Ren et al., 2016). Fusarium seedling blight can cause extensive damage to growing seedlings or foot rot later during the growing season, leading to reduced emergence and crop establishment and consequently yield losses in wheat (Wiese, 1987; Antalová et al., 2020). Moreover, FSB can provide a pathogen source for following FHB infection, creating reddish scabby spikes (Haigh et al., 2009; Li X. et al., 2010). Due to the global climate change and tillage management, FSB and FHB usually reach epidemic levels, causing huge yield losses across millions of hectares in global wheat production regions (Cheng et al., 2015; Liu et al., 2016). In addition, both FSB and FHB produce various mycotoxins during infection, with high toxicity, posing a threat to people as well as livestock (Pestka and Smolinski, 2005; Liu et al., 2012).

China is the largest producer and consumer of wheat (Shi and Ling, 2018). Cultivars play a major role in national wheat production, and developing and using resistant cultivars can confer protection to Fusarium pathogens. The analysis of the probable association between FSB and FHB can help develop new strategies to combat the Fusarium disease complex. Twelve Polish spring wheat cultivars and 18 spring wheat accessions from CIMMYT were examined for resistance to FSB and FHB by applying a highly aggressive fungal isolate, and no correlation was found between the two resistance types (Wisniewska and Busko, 2005). No significant correlation was also detected between FSB infection and FHB index and between FSB infection and DON content in a Wuhan/Nyubai doubled haploid (DH) wheat population (Somers et al., 2003; Tamburic-Ilincic et al., 2009). In subsequent research, QTLs for Fusarium resistance at seedling and spike stages were different, but further verification was required for various wheat populations (Tamburic-Ilincic et al., 2009). Comparatively, there have also been reports concerning the positive association between FSB and FHB resistance (Mesterhazy, 1987; Wu et al., 2005; Shin et al., 2014). Mesterhazy (1987) found a significant correlation between FSB and FHB resistance, and the most resistant genotypes at the seedling stage could yield the FHB resistant material with a large probability. By inoculation of wheat coleoptiles with *Fusarium graminearum* isolates, Wu et al. (2005) found a significant correlation between FSB and FHB resistance in the same genotype in the field. Using the clip-dipping inoculation method, Shin et al. (2014) found the remarkable correlation between the lesion length and Type II FHB resistance and suggested that the method for the evaluation of FSB resistance may provide a simple and feasible way for the early screening of FHB resistance in wheat.

Using linkage analysis, quite a few QTLs associated with FHB resistance were detected in 21 wheat chromosomes reported (Buerstmayr et al., 2009; Ma et al., 2020), with 7 FHB genes (*Fhb1*-*Fhb7*) being formally cataloged (Zhu et al., 2020). But FSB has not received much attention so far, and there have been very few studies on the QTLs for FSB resistance. A QTL on chromosome 5B controlling FSB resistance was identified in a DH population, and its linked marker WMC75 interpreted 13.8% of the phenotypic variation (Tamburic-Ilincic et al., 2009). Single major QTLs for FSB resistance, caused by *Microdochium nivale* and *Microdochium majus*, were detected on the chromosomes 1AL and 2BS, respectively (Ren et al., 2016).

Genome-wide association studies (GWAS) on the basis of linkage disequilibrium (LD) offer several advantages over linkage mapping, which has gained success in the analysis of different quantitative characteristics in wheat (Sapkota et al., 2019; Hu et al., 2020). For example, using 166 elite wheat varieties from Yellow and Huai River Valleys Wheat District in China, 120 common loci were detected for their associations with grain yield, among which 78 were potentially new (Li et al., 2019). In our previous studies, five QTLs were identified for their consistent associations with FHB resistance in a natural population, among which the QTLs on 5AS, 5AL, and 7DS were possibly new (Zhu et al., 2020). However, GWAS to identify FSB in wheat has not been reported yet, and the molecular mechanisms for FSB remain poorly understood.

In the present study, we evaluated FSB resistance in Chinese elite wheat lines and then performed GWAS and QTL analyses. The study aimed to (1) identify wheat germplasms with FSB resistance that could be used as resistance donors in breeding and confirm the relationship between FSB and FHB resistance caused by *Fusarium* pathogens, (2) uncover novel FSB-resistant loci that could be used in molecular marker-assisted breeding. The findings provide an insight into the genetics of FSB response in Chinese cultivars, and the developed markers associated with the mapped QTLs may be used for breeding FSB resistance wheat.

## Materials and Methods

### Plant Materials

A total of 240 common wheat cultivars or elite lines (Supplementary Table 1) were selected as the natural population to evaluate FSB resistance and perform GWAS analysis, as described in our previous study (Zhu et al., 2020). The population included 229 elite wheat cultivars (lines) developed in the main wheat-growing areas of China, covering 12 provinces with five agroecological systems, and could represent the current situation of breeding in China. The remaining 11 genotypes belonged to CIMMYT (10) and Australian (1). Seeds were harvested in the Wuhan Nanhu farm of Hubei Academy of Agricultural Sciences (N 30.28°, E 114.19°) during the cropping seasons in 2018–2019.

### Phenotyping

Wheat coleoptiles at the seedling stage were inoculated with conidiospores using the previously described method (Li X. et al., 2010; Cheng et al., 2015) with minor alterations. For seedling inoculation, the concentration of the macroconidia suspension for the aggressive isolate *F. graminearum* Huanggang 1 (Zhu et al., 2020) was regulated to 5 × 10^5^ spores/mL with sterilized distilled water. Forty full wheat seeds per cultivars (lines) were disinfected using 0.1% HgCl_2_ for 1 min and then rinsed twice using sterilized distilled water. Sterilized seeds were placed on wet filter paper in Petri dishes and incubated at 20°C in the dark for 2 days. Then 20 seeds with steady growth were picked, transferred to a sterilized germination box (length, width, and height of 11.5, 11.5, and 9.8 cm, respectively) with three layers of wet filter papers, and kept in the dark at 20°C for 1 day. Top coleoptiles (2–3 mm) were dissected, and a 3-μL aliquot of the macroconidial suspension was injected into the slant side of the dissected seedlings. Inoculated seedlings were stored in the germination box in the dark at 20°C, in dark as previously mentioned. The brown lesions of diseased seedlings were measured at 7 day post inoculation, and the lesion length was determined as previously described (Li X. et al., 2010). For each genotype, 20 wheat seedlings were examined each time, and the average value was used for subsequent analysis. The experiments were performed independently with triple replications.

### Statistical Analysis

The *t*-tests, as well as Pearson’s correlation analysis for independent samples, were conducted using IBM SPSS Statistics version 19.0 (IBM Corporation, Armonk, NY, United States). Histograms showing the distribution of the lesion length (cm) of 240 cultivars (lines) were made for each replicate with a script executed in R version 3.5.1.^1^

### Genotyping

Illumina 90 K SNP array genotyping was performed on 240 wheat accessions (Wang et al., 2014). Calling and filtering for SNPs, kinship, and population structure analysis have all been all elaborately explained in the previous research (Zhu et al., 2020). A total of 19,803 with MAF of >5 and <20% missing data of 22,922 polymorphic SNP_*S*_ were employed for subsequent analysis (Zhu et al., 2020). Population structure analysis was performed via ADMIXTURE.^2^ The population fell into three subgroups, basically based on geographic origin and pedigree (Zhu et al., 2020).

### Genome-Wide Association Studies for Fusarium Seedling Blight Resistance

Associations between genotypic and phenotypic data were analyzed in Tassel v5.0. A kinship (K) + PCA model was used to perform the MLM analysis for controlling the background variation and eliminating spurious marker-trait associations (MTAs). *R*^2^ exhibiting the variation explained by SNP was documented (Bradbury et al., 2007). SNPs with an adjusted-log_10_ (*P*-value) of ≥3.0 were considered associated with FSB resistance. The remarkable loci in a minimum of two repetitions detected in the research were stable QTLs. Remarkable SNP markers in one linkage disequilibrium on the same chromosome represented one locus.

### Kompetitive Allele-Specific PCR Assay

The SNP markers remarkably associated with FSB resistance were identified using GWAS and transformed into Kompetitive Allele-Specific PCR (KASP) markers to facilitate their application in MAS. The SNP contextual sequences were obtained at GrainGenes^3^ and the primers were designed by PolyMarker^4^ or the primer premier 5.0 (PREMIER Biosoft International, Palo Alto, CA, United States). Amplification was performed initially at 95°C for 15 min, 10 cycles of touchdown PCR (at 95°C for 20 s; an initial touchdown at 65°C, followed by a reduction of −1°C per cycle for 25 s), and the final 30 additional cycles for annealing (95°C for 10 s; 60°C for 60 s). Fluorescence signals were inspected under the multifunctional microplate reader PHERAstarPlus (BMG LABTECH, Ortenberg, Germany) and determined via KlusterCaller (LGC Genomics, Teddington, United Kingdom).

### Candidate Gene Analysis

To identify the candidate genes associated with typical SNPs, physical positions of markers before the chromosome name were introduced into Ensembl,^5^ and genes within a 2 Mb distance from typical SNPs were detected to assess their candidacy for FSB resistance. The transcript IDs of all these genes were obtained from wheat sequences (Alaux et al., 2018). We used another publicly available database, expVIP,^6^ to obtain the expression profiles of all these genes in wheat seedling coleoptile organs infected by *Fusarium* spp. (Ma et al., 2014; Powell et al., 2017). To visualize the expression profiles, heat maps were drawn using TBtools (Chen et al., 2018) from differently expressed genes, with the absolute value of log_2_ fold change of ≥1 or ≤–1 at either time point. Up-regulated genes in resistant varieties associated with disease resistance annotated by RefSeq Annotation v1.1 (Appels et al., 2018) were identified as candidate genes. The candidate genes were further used for searing the sequences with high similarity via NCBI, combined with a basic local alignment search tool (BLAST).^7^

## Results

### The Evaluation of Fusarium Seedling Blight Resistance

**FIGURE 1:**
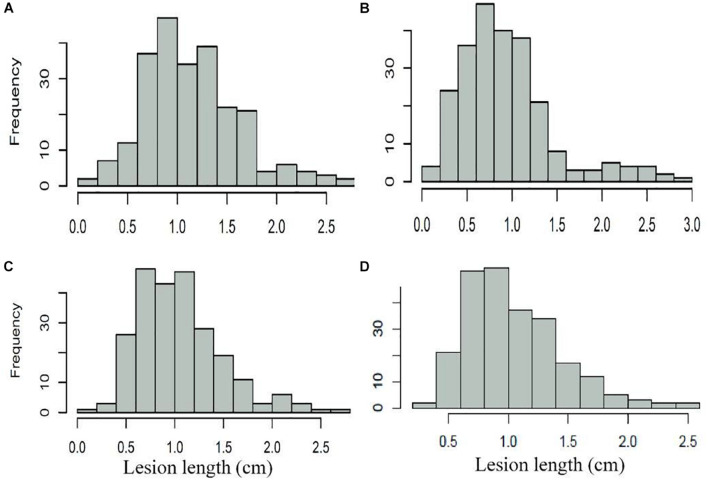
Frequency distribution of Fusarium seeding blight of 240 wheat cultivars (lines). **(A)** FSB_Rep1; **(B)** FSB_Rep2; **(C)** FSB_Rep3; and **(D)** FSB_mean.

The assessment of FSB resistance in 240 wheat accessions showed a lesion length within the range of 0.075–2.896, with a normal distribution in three replications (Table 1 and Figure 1). Pearson’s correlation coefficients among three replications ranged from 0.535 to 0.577 with a significant difference (*P* < 0.01), and the average values were significantly associated with repeats, with correlation coefficients of 0.826, 0.857, and 0.828 for each repeat (Table 1). Further analysis indicated that 23 accessions, including the elite cultivars Zhoumai 17, Yanzhan 4110, Yunong 035, Jimai 38, Yumai 69, Jingfumai 1, Zhoumai 16, Yannong 24, and Yangmai 11 showed an average lesion length of less than 0.6, with a potential for breeding for FSB resistance in wheat. There were 105, 71, 29, and 12 accessions showing the average lesion lengths within the ranges of 0.6–1.0, 1.01–1.40, 1.41–1.80, and >1.80, respectively. Representative accessions with different grades of resistance to FSB are represented in Table 2.

**TABLE 1:** Descriptive statistics and correlation coefficients of Fusarium seeding blight and Fusarium head blight of 240 wheat cultivars (lines).

	*T*-TEST					Correlations			
	Mean	Min	Max	SD	Std Error	FSB_Rep1	FSB_Rep2	FSB_Rep3	FSB_Mean
FSB_Rep1	1.159	0.139	2.769	0.478	0.031				
FSB_Rep2	0.946	0.075	2.896	0.531	0.034	0.535**			
FSB_Rep3	1.052	0.165	2.735	0.437	0.028	0.545**	0.577**		
FSB_Mean	1.049	0.346	2.510	0.404	0.026	0.826**	0.857**	0.828**	
BLUE of FHB^*a*^	46.66	5.00	89.00	16.571	1.070	–0.239**	–0.160*	–0.273**	–0.263**

**Significant at P < 0.01.

*Significant at P < 0.05.

*^a^Data from our previous study (Zhu et al., 2020).*

**TABLE 2:** Materials with different resistance levels to Fusarium seedling blight (FSB) (only representative materials are shown).

Mean of lesion length (cm)	Number	Representative cultivars (lines)
≤0.60	23	Zhengyumai 9987, Zhoumai 17, Yanzhan 4110, Yunong 035, Lianmai 2, Xikemai 4, Yumai 70–36, Zhongmai 1, Yan 2415, Luohan 2, Luomai 21, Jimai 38, Yumai 69, Jingfumai 1, Zhoumai 16, Yannong 24, and Yangmai 11
0.60–1.00	105	Xinmai 20, SYN1, Kenong 199, Zhongyu 10, Yangmai 22, Ningmai 13, Chuanmai 42, Zhengmai 366, Xinong 9871, Yangmai 13, Liangxing 99, Xinong 979, Jimai 22, Ocoroni, Mianmai 37, Xiaoyan 22, Yangmai 17, Ningmai 9, Hengguan 35, and Emai 27
1.01–1.40	71	Lantian 18, Jingdong 17, Emai 23, Yangmai 12, Xinmai 11, Mayoor, and Huaimai 20 Zhongmai 9, Zhengmai 9023, Jimai 20, Ningdong 10, 04 Zhong 36, Aikang 58, Chuanmai 50, Wuhan 1, Zhenmai 168, Ningchun 43, Jingmai 103, Lumai 21, Ningmai 16, Pingan 6, and Emai 580
1.41–1.80	29	Yumai 48, Emai 12, Een 6, Chuanmai 51, Yangmai 16, Lantian 23, Ningdong 11, Yangmai 158, Ningmai 8, Emai 18, Lunxuan 987, Xiangmai 25, Jingdong 8, and Xiaoyan 6
>1.80	12	Xinong 88, Jingzhou 66, Ningmai 11, Ning 7840, Zhongnong 2, Xiangmai 55, Jining 16, Een 5, Een 1, Sumai 3, and Gamenya

### The Correlation Between Fusarium Seedling Blight and Fusarium Head Blight

Correlation coefficients were determined based on FSB infection values in the research and BLUE values of FHB indices, calculated within 4 years, according to the results of our previous study (Zhu et al., 2020) using the same population. The average FSB lesion length was negatively correlated with the BLUE FHB index across the population, although a low coefficient of *R* = –0.263 was determined (Table 1). The most notable cultivar Sumai3 and its derivative Ning7840 with a high FHB resistance showed quite low resistance to FSB in this assay. Conversely, the FHB susceptible cultivars Zhengyumai 9987 and Zhoumai 17 (Zhu et al., 2020) showed relatively high resistance to FSB. However, some accessions such as Jingfumai 1 and Yangmai 11 had relatively high resistance to both FSB and FHB simultaneously (Zhu et al., 2020).

### Marker-Trait Association Analysis

**FIGURE 2:**
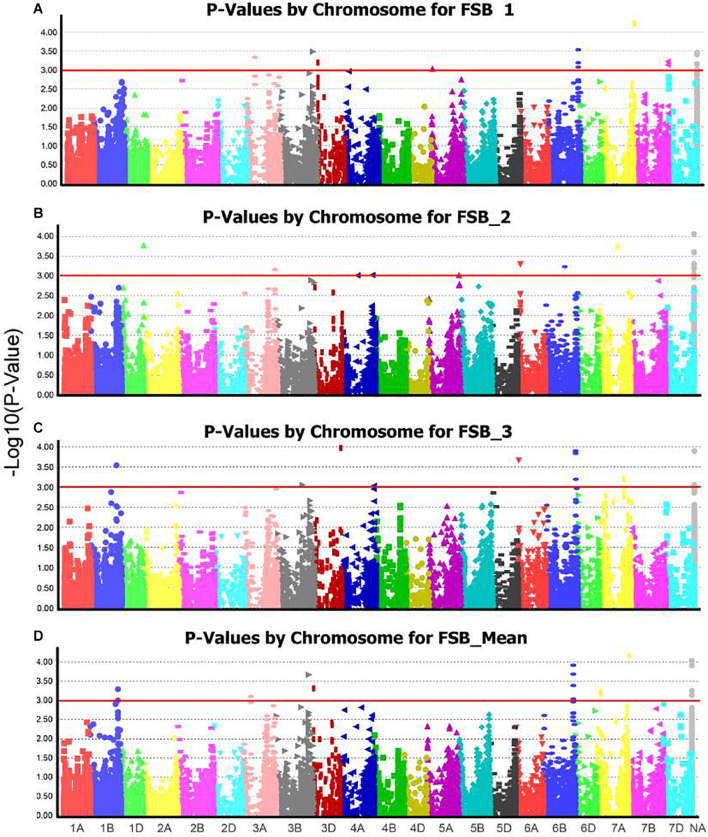
Manhattan plots from genome-wide association scan for Fusarium seedling blight (FSB) severities among 240 wheat accessions in **(A)** FSB_Rep1, **(B)** FSB_Rep2, **(C)** FSB_Rep3, and **(D)** FSB_mean. Dashed red horizontal line is the significant threshold level.

Six QTLs on chromosome arms 1DL, 3AS, 3BL, 6BL, 7AL, and Un, designated as Q*fsb.hbaas-1DL*, Q*fsb.hbaas-3AS*, Q*fsb.hbaas-3BL*, Q*fsb.hbaas-6BL*, Q*fsb.hbaas-7AL*, and *Qfsb.hbaas-un*, respectively, were significant in a minimum of two repetitions, interpreting phenotypic variation of 4.83–7.53% (Table 3 and Figure 2). Representative significant markers for these QTLs were IWB41243, IWB64668, IWB3107, IWA3221, IWB41907, and IWB36312, respectively. For the goal of identifying minor QTL, this study was underpowered because of small population size which results in not seeing high signals.

**TABLE 3:** Loci significantly associated with FSB resistance in at least two environments in the 240 wheat cultivars (lines) using the mixed linear model (MLM) model in Tassel v5.0.

QTL	Marker^*a*^	Variant^*b*^	Chr^*c*^	Position (Mb)^*d*^	Environment	*P*-value	*R*^2^ (%)^*e*^
*Qfsb.hbaas-1DL*	*IWB41243*	A/G	1DL	458.9	Rep2/Mean	6.36E-04/7.37E-04	5.74/5.33
*Qfsb.hbaas-3AS*	*IWB64668*	T/G	3AS	176.6	Rep1/Mean	4.57 E-04/8.16 E-04	5.12/4.83
*Qfsb.hbaas-3BL*	*IWB3107*	G/A	3BL	723.0	Rep1/Mean	3.24 E-04/2.14 E-04	5.47/6.29
*Qfsb.hbaas-6BL*	*IWA3221*	C/T	6BL	668.0	Rep1/Rep3/Mean	6.34E-04/6.39E-04/1.21 E-04	5.07/5.20/6.50
*Qfsb.hbaas-7AL*	*IWB41907*	G/A	7AL	724.1	Rep1/Mean	5.86 E-05/6.99 E-05	7.00/7.53
*Qfsb.hbaas-un*	*IWB36312*	A/C	Un	32.2	Rep2, Mean	2.51 E-04/9.40 E-05	6.18/6.74

*^a^Representative markers showing the strongest association with the FSB resistance locus.*

*^b^Favorable allele is underlined.*

*^c^Chr, chromosome.*

*^d^Physical positions based on the Chinese Spring reference genome sequences from the International Wheat Genome Sequencing Consortium (IWGSC, http://www.wheatgenome.org).*

*^e^Percentage of phenotypic variance explained.*

Of the 240 genotypes, 12, 180, 130, 204, 220, and 217 possessed the resistance alleles Q*fsb.hbaas-1DL*, Q*fsb.hbaas-3AS*, Q*fsb.hbaas-3BL*, Q*fsb.hbaas-6BL*, Q*fsb.hbaas-7AL*, and *Qfsb.hbaas-un*, respectively, on the basis of marker analysis (Table 4 and Supplementary Table 1). The mean FSB lesion length in accessions with favorable *Qfsb.hbaas-1DL* alleles was 21.4% shorter than with unfavorable alleles. Discrepancies between *Qfsb.hbaas-6BL* and *Qfsb.hbaas-7AL* were much greater (31.7 and 36.3%, respectively). In *Qfsb.hbaas-3AS*, *Qfsb.hbaas-3BL*, and *Qfsb.hbaas-un*, the FSB lesion lengths were reduced by 17.4, 13.8, and 8.8%, respectively (Table 4).

**TABLE 4:** *T-*tests for differences in Fusarium seedling blight between two groups of wheat accessions with contrasting resistance or susceptibility alleles for quantitative trait loci (QTL) on chromosomes 1D, 3A, 3B, 6B, 7A, and Un.

QTL	Present/Absent^#^	Number	Rep1	Rep2	Rep3	Mean
*Qfsb.hbaas-1D*	Present	12	1.02a	0.57a	0.78a	0.79a
	Absent	223	1.15a	0.95b	1.06b	1.06b
*Qfsb.hbaas-3A*	Present	180	1.07A	0.89A	1.03a	1.00A
	Absent	58	1.39B	1.11B	1.14a	1.21B
*Qfsb.hbaas-3B*	Present	130	1.05A	0.84A	0.94A	0.94A
	Absent	108	1.26B	1.06B	1.17B	1.16B
*Qfsb.hbaas-6B*	Present	204	1.08A	0.88A	1.00A	0.99A
	Absent	31	1.56B	1.40B	1.41B	1.45B
*Qfsb.hbaas-7A*	Present	220	1.10A	0.89A	1.02A	1.00A
	Absent	15	1.69B	1.48B	1.54B	1.57B
*Qfsb.hbaas-un*	Present	217	1.15a	0.92a	1.05a	1.04a
	Absent	19	1.15a	1.14a	1.14a	1.14a

*^#^The QTL present superior effect (present) or inferior effect (absent), A and B represent significant at P < 0.01, a and b represent significant at P < 0.05.*

### The Relationship Between the Fusarium Seedling Blight Lesion Length and the Number of Favorable Alleles

**FIGURE 3:**
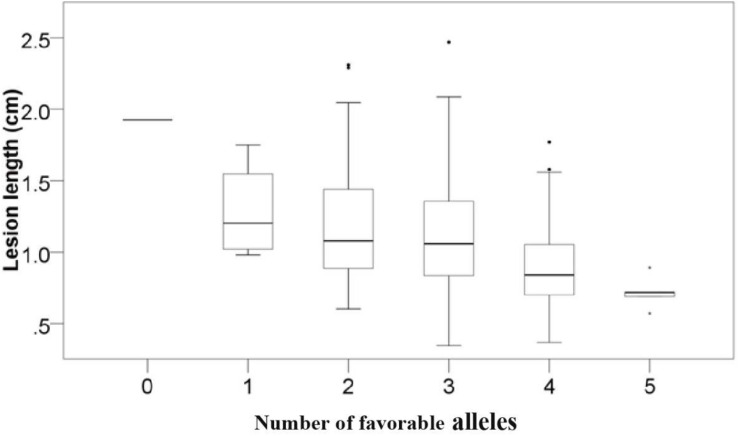
Relationship between the number of favorable quantitative trait locis (QTLs) and the mean FSB severity. Lesion length indicates the FSB severity.

To examine the pyramiding effects of favorable alleles of various QTLs, we analyzed the number of favorable alleles in 6 mapped loci per accession. Favorable alleles were 0–5. Linear regression (*r*^2^ = 0.872) revealed the correlation between disease severity and the number of favorable alleles (Figure 3 and Supplementary Table 2). Accessions including a larger number of favorable alleles, such as Zhoumai16 (5), Xikemai4 (4), and Zhoumai17 (4), exhibited strong FSB resistance. Conversely, Yang 07–15, with no favorable alleles, exhibited low FSB resistance (Supplementary Table 1).

### Development of Kompetitive Allele-Specific PCR Markers for Quantitative Trait Locis Underlying Resistance to Fusarium Seedling Blight

The SNPs (IWB41243, IWB64668, IWB3107, and IWA3221), associated with Q*fsb.hbaas-1DL*, Q*fsb.hbaas-3AS*, Q*fsb.hbaas-*3BL, and Q*fsb.hbaas-6BL*, respectively, were successfully used to develop KASP markers (Table 5). All 240 wheat accessions were genotyped by these KASP markers. The results demonstrated that the genotypes from the KASP test were identical to the chip assay with low-frequency oscillations (2.5, 5.0, 3.3, and 3.8% for each marker, respectively).

**TABLE 5:** Primer sequences of single nucleotide polymorphism (SNP) markers for validation in wheat lines by Kompetitive Allele-Specific PCR (KASP) assay.

QTL	Primer	Sequence (5′–3′)
*Qfsb.hbaas-1D*	P41243A	CCACCTTTCAACTCGCTCA
	P41243B	CCACCTTTCAACTCGCTCG
	P41243C	CTCACTTCTTCTAGAACAAATCGAA
*Qfsb.hbaas-3A*	P64668A	TGCAATCTTGGACAAACATCAT
	P64668B	TGCAATCTTGGACAAACATCAG
	P64668C	GTGCTTTGTCAACAACAGATGC
*Qfsb.hbaas-3B*	P3107A	GGTCGCATCAGGAAGAGCA
	P3107B	GGTCGCATCAGGAAGAGCG
	P3107C	TTCTTCCCTTTACAGACTCTTCAGC
*Qfsb.hbaas-6B*	P3221A	GTTTTTGTGGCTGCGGGT
	P3221B	GTTTTTGTGGCTGCGGGC
	P3221C	TTCTTCCCTTTACAGACTCTTCAGC

*A Primer labeled with FAM: GAAGGTGACCAAGTTCATGCT. B Primer labeled with HEX: GAAGGTCGGAGTCAACGGATT.*

### The Prediction of Candidate Genes

**FIGURE 4:**
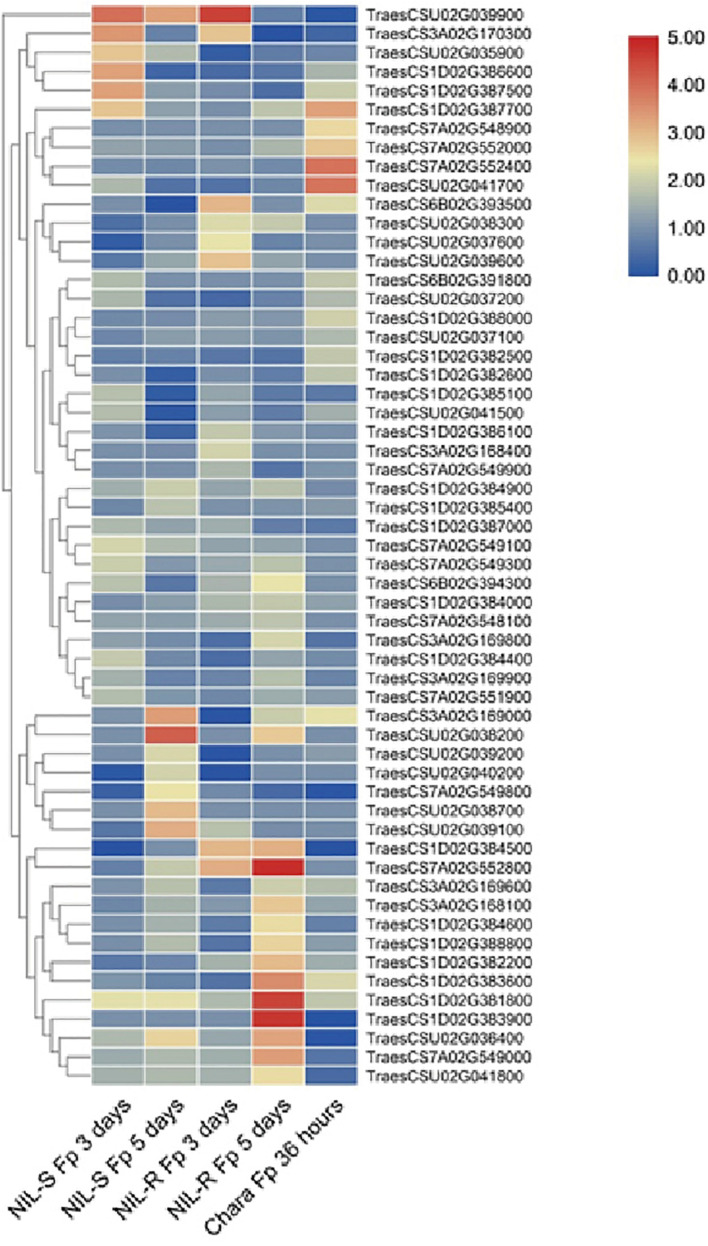
Clustering heatmap for differentially expressed genes infected by *Fusarium* spp. compared to those in the mock. S-NIL1-Fp 3 days, susceptible isolines infected by *Fusarium* spp. after 3 days; S-NIL1-Fp 5 days, susceptible isolines infected by *Fusarium* spp. after 5 days; R-NIL1-Fp 3 days, resistant isolines infected by *Fusarium* spp. after 3 days; R-NIL1-Fp 5 days, resistant isolines infected by *Fusarium* spp. after 3 days; Chara-Fp 36 h, wheat culture “Chara” infected by *Fusarium* spp. after 36 h.

A total of 291 candidate genes were located within the candidate regions. Combined with transcriptome data from public databases (Ma et al., 2014; Powell et al., 2017), 57 genes were differently expressed in wheat seedling coleoptile organs after infection by *Fusarium* spp. (Figure 4 and Supplementary Table 3). Among them, eight unique annotated genes involved in plant disease resistance were considered as candidates for mapping QTLs (Table 6). Two genes encoding the disease resistance protein RPM1 and receptor-like protein kinase were identified as candidates for *Qfsb.hbaas-1DL*. A gene encoding L-type lectin receptor kinase might contribute to FSB resistance for Q*fsb.hbaas-3AS*. A gene encoding MADS-box protein was considered as a candidate for Q*fsb.hbaas-6BL*. For *Qfsb.hbaas-7AL*, a gene encoding NAC domain-containing protein was identified. Three genes encoding serine/threonine kinase-like protein, HCBT-like defense response protein, and subtilisin-like protease might contribute to FSB resistance for *Qfsb.hbaas-un*.

**TABLE 6:** Candidate genes for Fusarium seeding blight resistance.

Gene ID	Chr^*a*^	Position^*b*^ (Mb)	Predicted function^*c*^	Identity (%)	Orthologous gene
TraesCS1D02G388800	1D	460.85	Disease resistance protein RPM1	99.40	LOC109754777
TraesCS1D02G381800	1D	457.07	Receptor-like protein kinase	100	LOC109748921
TraesCS3A02G169600	3A	177.70	L-type lectin receptor kinase	98.82	LOC109777203
TraesCS6B02G391800	6B	666.77	MADS-box protein	99.36	LOC119321270
TraesCS7A02G549000	7A	723.26	NAC domain-containing protein	90.58	LOC109760823
TraesCSU02G041800	Un	34.16	Serine/threonine kinase-like protein	83.01	LOC109786647
TraesCSU02G041700	Un	34.14	HCBT-like defense response protein	92.54	LOC109754238
TraesCSU02G039600	Un	32.13	Subtilisin-like protease	87.27	LOC109786891

*^a^Chr, chromosome.*

*^b^Gene annotations were referred to IWGSC Ref Seq annotation v1.1 (IWGSC, http://www.wheatgenome.org/).*

*^c^The sequences of T. aestivum gene were blasted in the NCBI (http://www.ncbi.nlm.nih.gov/), databases to identify putative gene functions.*

## Discussion

Breeding the cultivars with resistance to FSB and FHB offers an efficient way to control complex diseases and decrease yield losses or mycotoxin occurrence in agricultural products. Combining the two resistance traits in one elite wheat cultivar is challenging due to its exposure to regulated independent genetic loci and also the restricted size of resistant germplasm in the natural environment (Tamburic-Ilincic et al., 2009). In this study, 229 elite Chinese wheat cultivars and lines, which represent the genetic diversity in newly assembled accessions in China (Jia et al., 2020), were investigated. A total of 54 cultivars reached the maximum annual acreage of 1 × 10^5^ ha during 2000–2016, and quite a few cultivars such as Liangxing 99, Zhoumai 18, Jimai 22, Zhoumai 22, Zhengmai 9023, and Aikang 58, have been used as founder parents in breeding programs (Jia et al., 2020). Among them, 23 accessions showed a relatively high-level resistance with an average lesion length of less than 0.6 cm, exhibiting the potential for breeding for FSB resistance in wheat. Despite the negative correlation between FSB infection and FHB index in the population (Table 1), combined with our previous research results (Zhu et al., 2020), we also found that some cultivars such as Jingfumai 1 and Yangmai 11 had relatively great resistance to both FSB and FHB simultaneously. Jingfumai 1 and Yangmai 11 both bred in the Middle and Lower Yangtze River Valleys were red-grained spring wheat and high resistant to pre-harvest sprouting. The spike length of the two lines was 8.0 and 8.4 cm, and the spikelet number was 18.3 and 17.2, respectively. The research conformed to the findings reported by Ren et al. (2015), who found that the FSB-resistant cultivar Petrus was simultaneously resistant to FHB. These lines were good parent candidates for future crosses in breeding for *Fusarium* seedling resistance and head blight resistance in wheat.

The correlation analysis between FSB and FHB resistance has been reported in previous studies (Mesterhazy, 1987; Gosman et al., 2005; Ruckenbauer et al., 2001; Tamburic-Ilincic et al., 2009; Shin et al., 2014). Few studies revealed a positive association between FSB and FHB resistance. Shin et al. (2014) reported the significant correlation coefficients between the lesion lengths and Type II resistance to FSB and FHB, but the number of samples was not very large and only 29 Korean winter wheat cultivars were chosen in trials. The CIMMYT spring wheat line LSP2 was proved to have a high susceptibility to FSB and resistance to FHB, caused by *Fusarium* spp. (Ren et al., 2016). The widely planted British winter wheat cultivar Rialto was highly resistant to FSB, caused by Microdochium spp., while some reports revealed its high susceptibility to FHB (Srinivasachary et al., 2008). Some wheat cultivars, including Chinese local cultivars Wangshuibai and Sumai3, were highly resistant to FHB, and high susceptibility to FSB was also found (Mesterhazy, 1987; Wu et al., 2005; Li X. et al., 2010). Using Sumai3 and Falat as the cultivars resistant and susceptible to FHB, respectively, Sorahinobar et al. (2016) observed little correspondence between wheat seedling tolerance to *F.* graminearum crude extract and resistance to FHB. In both our previous (Zhu et al., 2020) and present studies, Sumai3 showed an FSB-susceptible reaction while exhibiting FHB resistance in response to *Fusarium* spp. Negative correlations, albeit low, between FSB and FHB resistance were observed in the present study. These results also agreed with the findings published by Bruins et al. (1993); Ruckenbauer et al. (2001), Gosman et al. (2005), and Tamburic-Ilincic et al. (2009), who discovered that greenhouse experiments in seedling cannot be used when selecting for FHB resistance.

Two transgenic wheat lines expressing two anti-fungal peptides exhibited enhanced resistance to FSB and FHB, while FHB resistance could be detected in the other five lines (Liu et al., 2012). Transgenic wheat overexpressing an *A. thaliana NPR1* gene could increase the severity of FSB, although FHB resistance increased simultaneously (Gao et al., 2013). Li X. et al. (2010) firstly reported a close association between FHB and FSB resistance in wheat using distinct molecular profiles for disease-associated gene expression and suggested that there may be two resistance mechanisms in wheat spikes and seedlings in response to FHB pathogens. Some studies have also shown different QTLs for resistance to FSB and FHB (Tamburic-Ilincic et al., 2009; Ren et al., 2016). In our previous study, five QTL on chromosome arms 1AS, 2DL, 5AS, 5AL, and 7DS were associated with FHB resistance, explaining 5.4–10.3% of phenotypic variation (Zhu et al., 2020). Using the same population, we identified six entirely different QTL on chromosome arms 1DL, 3AS, 3BL, 6BL, 7AL, and Un, interpreting phenotypic variation of 4.83–7.53% (Table 3 and Figure 2). Different regions suggested the differences in QTLs for resistance to FSB and FHB and resistance to FSB and FHB is probably independent. Thus, due to gene recombination, a few accessions in this research exhibited Fusarium resistance in seedling and head.

There might be two main reasons why discrepant alterations in genes or QTLs could be associated with resistance to both FSB and FHB. The first reason is the infection time; FSB infection occurs during the seedling growth, whereas FHB infection occurs during the flowering stage. It is well known that different genes can be involved in the resistance of host plants to one disease in various stages of plant development (Li H. B. et al., 2010). The second reason is the infection of different organs by the two diseases. Miedaner (1997) put forward the idea that complex interactions can occur between the resistance to diseases across different plant growth stages, plant organs, or host genotypes. We suggested that the mechanisms and genes involved in resistance to Fusarium during seedling growth and spike formation are possibly different, and separate screening is essential to evaluate the resistance to FSB and FHB, caused by *Fusarium* in breeding programs.

The discovery of novel genes or QTLs is a constant challenge and extremely important in wheat breeding. Many QTLs associated with FHB resistance have been detected (Zhu et al., 2020), whereas there have been very few studies on QTLs for resistance to FSB. Using a Wuhan/Nyubai doubled haploid (DH) wheat population, merely one QTL, controlling FSB resistance, detected on chromosome 5B and marker WMC75, could interpret 13.8% phenotypic variation in the trait (Tamburic-Ilincic et al., 2009). Using the Rialto/LSP2 DH population, a single major QTL conferring FSB resistance to *Microdochium majus* was found on the chromosome 1AL in all four experiments, accounting for 32.5–56.6% of the phenotypic variation; a significant QTL conferring FSB resistance to *Microdochium nivale* was discovered on chromosomes 2BS and explained 29.3–55.0% of the phenotypic variation (Ren et al., 2016). In this research, we detected six QTLs on chromosome arms 1DL, 3AS, 3BL, 6BL, 7AL, and Un that were significant for resistance to FSB and previously uncharacterized in wheat, and therefore they were likely to be novel QTLs for FSB resistance. The average lesion length dramatically decreased when the number of favorable alleles increased (Figure 3). This finding suggested the prospective role of these QTLs in FSB resistance. But some QTls such as *Qfsb.hbaas-1D* and *Qfsb.hbaas-7A* have very tiny minor allele frequencies, they may not be real signals. To validate the real effects of these 6 mapped QTLs, typical significant markers should be examined using various bi-parental and natural populations.

Combined with the analysis of transcriptome data, we identified eight unique annotated genes involved in plant disease resistance in wheat in IWGSC RefSeq v1.1, which were linked to the six QTLs (Table 6). The RPM1 is a CC-NB-LRR protein that conferred resistance against *Pseudomonas syringae pv. maculicola 1* (Mackey et al., 2002; Su et al., 2017), and TaRPM1 might play a key part in the wheat innate immune response to the infection caused by the powdery mildew pathogen (Nie and Ji, 2019). Receptor-like protein kinases, which are the largest gene family in plants, play essential roles in combating infection caused by pathogens (Liu et al., 2017). TaCRK2, a novel receptor-like kinase gene, plays a positive role in resistance to leaf rust in wheat through the regulation of the HR cell death process induced by *P. triticina* (Gu et al., 2020). L-type lectin receptor kinases are omnipresent in plants and play an important role in the initiation of innate immunity (Wang and Bouwmeester, 2017). An L-type lectin receptor kinase in *Haynaldia villosa* conferred powdery mildew resistance in wheat (Wang et al., 2018). Moreover, MIKC-type MADS-box genes exhibited new expression patterns in response to biotic stress (Schilling et al., 2020). The NAC protein constituting the most important plant transcription factors could enhance resistance to Fusarium head blight, as well as stripe rust (Ning et al., 2010; Perochon et al., 2019). Serine/threonine kinase, one of the largest protein kinase gene families, could confer resistance to powdery mildew and stripe rust in wheat (Cao et al., 2011; Gou et al., 2015). HCBT-like defense response protein, which was rapidly and transiently expressed after being induced by the pathogen, plays an essential role in fungal pathogen resistance (Brooks et al., 2002). The subtilisin-like protease is associated with pathogenicity in fungi and plays an important role in resistance to leaf rust in wheat (Fan et al., 2016).

## Data Availability Statement

The original contributions presented in the study are included in the article/Supplementary Material, further inquiries can be directed to the corresponding authors.

## Author Contributions

YL, DM, and CG guided the design of the experiment. YL, GZ, ZZ, LC, HN, WH, HT, JS, and YZ directed the data analysis. YL and GZ conducted data analysis and wrote the manuscript. DM and CG supervised the experiment and confirmed the manuscript. YL was the guarantor of this work, so she could have full access to all the data in the research and responsible for the integrity of the data and the accuracy of the data analysis. All authors contributed to the article and approved the submitted version.

## Conflict of Interest

The authors declare that the research was conducted in the absence of any commercial or financial relationships that could be construed as a potential conflict of interest.

## Publisher’s Note

All claims expressed in this article are solely those of the authors and do not necessarily represent those of their affiliated organizations, or those of the publisher, the editors and the reviewers. Any product that may be evaluated in this article, or claim that may be made by its manufacturer, is not guaranteed or endorsed by the publisher.

## Footnotes

1
www.r-project.org

2
http://software.genetics.ucla.edu/admixture

3
https://wheat.pw.usda.gov/GG3/

4
http://polymarker.tgac.ac.uk

5
https://urgi.versailles.inra.fr/gb2/gbrowse/wheat_survey_sequence_annotation

6
http://www.wheat-expression.com/

7
http://blast.ncbi.nlm.nih.gov/Blast.cgi
