# Supplementary material for: Investigation and Genome-Wide Association Analysis of Fusarium Seedling Blight Resistance in Chinese Elite Wheat Lines
Source: Front Plant Sci. 2021 Nov 17;12:777494. doi: 10.3389/fpls.2021.777494 (PMC8635748; doi:10.3389/fpls.2021.777494)
Supplement: Supplementary file 1 [file Data_Sheet_1.docx]

Table S1 Putative QTL and mean values of FSB of the 240 wheat accessions.

| No. | Cultivar | *Qfsb.hbaas-1D* | *Qfsb.hbaas-3A.1* | *Qfsb.hbaas-3B* | *Qfsb.hbaas-6B* | *Qfsb.hbaas-7A* | *Qfsb.hbaas-un* | FSB_Mean(cm) |
| --- | --- | --- | --- | --- | --- | --- | --- | --- |
| 1 | Beijing 0045 | - | - | + | + | - | + | 1.326 |
| 2 | Jingdong 8 | - | - | - | - | - | + | 1.749 |
| 3 | Jingdong 17 | - | - | - | + | - | + | 1.005 |
| 4 | Lunxuan 987 | - | - | - | + | + | + | 1.675 |
| 5 | Zhongmai 9 | - | - | + | - | - | - | 1.063 |
| 6 | Zhongmai 12 | - | - | - | + | - | + | 1.092 |
| 7 | Zhongnong 2 | - | + | - | + | - | + | 2.024 |
| 8 | Wanmai 38 | - | + | + | + | - | + | 0.646 |
| 9 | Wanmai 50 | - | + | + | + | - | + | 0.762 |
| 10 | Wanmai 52 | - | + | + | + | - | + | 0.932 |
| 11 | Han 3475 | - | + | + | + | - | + | 1.051 |
| 12 | Han 5316 | - | + | - | + | - | - | 1.233 |
| 13 | Han 6172 | - | + | + | - | - | + | 0.889 |
| 14 | Heng 115 | - | + | - | + | - | + | 0.916 |
| 15 | Heng 136 | - | - | - | + | - | + | 1.052 |
| 16 | Heng 4422 | + | - | - | - | - | + | 0.636 |
| 17 | Hengguan 35 | + | + | - | + | - | + | 0.850 |
| 18 | Jimai 30 | - | + | + | + | - | + | 0.898 |
| 19 | Jimai 38 | - | - | + | + | - | + | 0.504 |
| 20 | Kenong 199 | - | + | - | + | - | + | 0.615 |
| 21 | Kenong 9204 | - | + | + | + | - | + | 0.922 |
| 22 | Shijiazhuang 8 | - | - | - | - | + | + | 1.066 |
| 23 | 04 zhong 36 | - | + | + | + | - | + | 1.196 |
| 24 | Aikang 58 | - | + | + | + | - | + | 1.216 |
| 25 | Aizao 781-99 selection | - | + | + | + | - | + | 0.700 |
| 26 | Bainong 160 | - | + | + | + | - | + | 0.543 |
| 27 | Fanmai 5 | - | - | + | + | - | + | 0.973 |
| 28 | Jinfeng 3 | - | + | - | + | - | - | 0.962 |
| 29 | Kaimai 18 | - | + | + | + | - | + | 0.766 |
| 30 | Keda 9612 | + | + | + | - | - | + | 0.771 |
| 31 | Luo 4-168 | - | + | + | + | - | + | 0.579 |
| 32 | Luohan 2 | - | + | - | + | - | + | 0.489 |
| 33 | Luohan 6 | - | + | - | - | - | - | 0.978 |
| 34 | Luohan 7 | - | + | - | - | - | - | 1.343 |
| 35 | Luomai 21 | - | + | + | + | - | + | 0.496 |
| 36 | Luomai 6010 | - | + | + | + | - | + | 0.967 |
| 37 | Luoxin 998 | + | + | + | + | - | + | 0.717 |
| 38 | Pingan 3 | U | + | - | + | - | - | 0.983 |
| 39 | Pingan 6 | - | + | + | + | - | + | 1.393 |
| 40 | Pumai 10 | - | + | + | + | - | + | 0.778 |
| 41 | Wenmai 6 | - | + | - | + | - | - | 0.790 |
| 42 | Wenmai 7 | - | + | - | + | - | - | 0.885 |
| 43 | Wenmai 18 | - | + | - | + | - | - | 1.549 |
| 44 | Wenmai 19 | - | + | - | + | - | - | 0.601 |
| 45 | Xiangmai 99 | - | + | + | + | - | + | 1.313 |
| 46 | Xinmai 11 | - | + | - | + | - | + | 1.034 |
| 47 | Xinmai 13 | + | + | - | + | - | + | 0.666 |
| 48 | Xinmai 16 | - | + | - | + | - | + | 0.973 |
| 49 | Xinmai 18 | - | + | + | + | - | + | 0.707 |
| 50 | Xinmai 19 | - | + | - | + | - | - | 0.795 |
| 51 | Xinmai 20 | - | + | + | + | - | + | 0.596 |
| 52 | Xinmai 22 | - | - | + | + | - | + | 0.845 |
| 53 | Xinmai 26 | - | + | + | + | - | + | 1.254 |
| 54 | Xinmai 208 | - | + | + | + | - | + | 0.559 |
| 55 | Xinmai 9817 | - | + | - | + | - | + | 0.936 |
| 56 | Xinmai 9817 selection | - | + | + | + | - | U | 1.084 |
| 57 | Xuke 1 | - | + | + | + | - | + | 0.751 |
| 58 | Yanzhan 4110 | - | + | + | + | - | + | 0.417 |
| 59 | Yumai 10 | - | - | + | + | - | + | 1.325 |
| 60 | Yumai 38 | - | + | + | + | - | + | 1.090 |
| 61 | Yumai 48 | - | + | - | - | - | + | 1.403 |
| 62 | Yumai 49-168 | - | + | - | + | - | - | 0.672 |
| 63 | Yumai 49-198 | - | + | - | + | - | - | 1.021 |
| 64 | Yumai 52 | - | + | - | + | - | + | 0.707 |
| 65 | Yumai 69 | - | + | + | + | - | + | 0.516 |
| 66 | Yumai 70 | - | + | + | + | - | + | 0.794 |
| 67 | Yumai 70-36 | - | + | + | + | - | + | 0.443 |
| 68 | Yunong 035 | - | + | + | + | - | + | 0.421 |
| 69 | Yunong 202 | - | + | - | + | - | + | 0.887 |
| 70 | Zhengmai 004 | - | - | - | + | - | + | 0.885 |
| 71 | Zhengmai 98 | - | + | + | - | - | + | 1.318 |
| 72 | Zhengmai 366 | - | + | + | + | - | + | 0.673 |
| 73 | Zhengmai 9023 | - | + | - | + | - | + | 1.063 |
| 74 | Zhengmai 9694 | - | + | + | + | - | + | 0.529 |
| 75 | Zhengnong 17 | - | + | + | + | - | + | 0.652 |
| 76 | Zhengyumai 958 | - | + | + | + | - | + | 0.735 |
| 77 | Zhengyumai 9987 | - | + | - | + | - | + | 0.346 |
| 78 | Zhongmai 1 | - | + | + | + | - | + | 0.468 |
| 79 | Zhongyu 10 | - | + | - | + | - | + | 0.627 |
| 80 | Zhoumai 16 | - | + | + | + | + | + | 0.571 |
| 81 | Zhoumai 17 | - | + | + | + | - | + | 0.368 |
| 82 | Zhoumai 18 | - | + | + | + | - | + | 1.000 |
| 83 | Zhoumai 22 | - | + | + | + | - | + | 0.837 |
| 84 | Zhoumai 23 | + | + | + | + | - | + | 0.688 |
| 85 | Huaimai 17 | - | + | + | + | - | + | 0.675 |
| 86 | Huaimai 18 | - | - | + | + | - | + | 1.076 |
| 87 | Huaimai 20 | - | - | + | + | - | + | 1.044 |
| 88 | Lianmai 1 | - | - | - | + | - | + | 1.092 |
| 89 | Lianmai 2 | - | - | + | + | - | + | 0.441 |
| 90 | Xumai 27 | - | + | - | + | - | + | 1.205 |
| 91 | Xumai 29 | - | + | - | + | - | + | 0.854 |
| 92 | Xumai 216 | - | + | - | + | - | - | 0.698 |
| 93 | Liken 2 | - | + | - | - | - | + | 1.597 |
| 94 | Qinnong 142 | - | + | U | U | - | + | 0.790 |
| 95 | Shaan 253 | - | + | + | + | - | + | 1.182 |
| 96 | Shaan 627 | - | + | - | + | - | + | 0.808 |
| 97 | Shaan 715 | - | + | + | + | - | + | 0.849 |
| 98 | Shaanmai 139 | - | + | + | + | - | + | 1.455 |
| 99 | Shaanmai 150 | - | + | + | + | - | + | 0.831 |
| 100 | Shaanmai 159 | - | + | + | U | - | + | 1.219 |
| 101 | Shaannong 78 | - | + | + | + | - | + | 0.913 |
| 102 | Shaannong 138 | - | + | - | + | - | + | 1.266 |
| 103 | Shaannong 757 | - | + | + | + | - | + | 0.955 |
| 104 | Xiaoyan 6 | U | - | - | + | U | U | 1.750 |
| 105 | Xiaoyan 22 | - | + | + | + | - | + | 0.786 |
| 106 | Xiaoyan 107 | - | + | + | + | - | + | 0.879 |
| 107 | Xiaoyan 166 | - | + | - | + | - | + | 0.829 |
| 108 | Xinong 88 | - | + | + | + | - | + | 1.769 |
| 109 | Xinong 979 | - | - | + | - | - | + | 0.754 |
| 110 | Xinong 2000 | - | + | + | + | - | + | 0.800 |
| 111 | Xinong 3517 | - | + | + | + | - | + | 1.207 |
| 112 | Xinong 9871 | - | + | - | + | - | + | 0.708 |
| 113 | Yuanfeng 175 | - | + | + | + | - | + | 0.947 |
| 114 | Hemai 13 | - | + | - | + | - | + | 1.486 |
| 115 | Jimai 19 | - | + | + | + | - | + | 0.714 |
| 116 | Jimai 20 | - | - | + | + | - | + | 1.107 |
| 117 | Jimai 21 | - | - | + | + | - | + | 1.356 |
| 118 | Jimai 22 | - | - | + | + | - | + | 0.756 |
| 119 | Jinan 17 | + | - | - | + | - | + | 0.907 |
| 120 | Jinan 17 selection | + | - | - | + | - | + | 0.983 |
| 121 | Jining 16 | - | + | - | + | - | + | 2.087 |
| 122 | Liangxing 99 | - | - | + | + | - | + | 0.730 |
| 123 | Lumai 21 | - | + | - | + | - | + | 1.340 |
| 124 | Lunong 116 | - | - | + | + | - | + | 1.022 |
| 125 | Qingfeng 1 | - | - | - | + | - | + | 0.699 |
| 126 | Shannong 15 | - | - | - | + | - | + | 1.352 |
| 127 | Shannong 16 | - | + | - | + | - | + | 1.282 |
| 128 | Shannong 189 | - | + | - | + | - | + | 0.957 |
| 129 | Shannong 664 | - | + | + | + | - | + | 0.800 |
| 130 | Shannong 8355 | - | + | - | + | - | + | 1.634 |
| 131 | Tainong 18 | - | - | + | + | - | + | 0.752 |
| 132 | Taishan 21 | - | + | - | + | - | + | 1.418 |
| 133 | Taishan 23 | - | + | - | + | - | + | 1.877 |
| 134 | Weimai 8 | - | - | + | + | - | + | 1.395 |
| 135 | Yan 2415 | - | + | - | + | - | + | 0.480 |
| 136 | Yan 5158 | - | - | - | + | - | + | 0.943 |
| 137 | Yan 5286 | - | + | + | + | - | + | 1.057 |
| 138 | Yannong 19 | - | - | + | + | - | + | 0.830 |
| 139 | Yannong 21 | - | + | + | + | - | + | 0.896 |
| 140 | Yannong 22 | - | + | - | - | - | + | 0.930 |
| 141 | Yannong 24 | - | - | + | + | - | + | 0.582 |
| 142 | Chang 6359 | - | + | + | - | - | + | 0.953 |
| 143 | Lin Y867 | - | + | + | + | - | + | 0.942 |
| 144 | Lin Y7287 | - | + | - | + | - | + | 1.218 |
| 145 | Linfen 137 | - | + | - | + | - | + | 0.728 |
| 146 | Linfen 138 | - | + | - | + | - | + | 0.967 |
| 147 | Linyou 2069 | - | + | + | + | - | + | 0.764 |
| 148 | E 07901 | - | + | + | + | - | + | 0.751 |
| 149 | Een 1 | - | - | - | - | + | + | 2.310 |
| 150 | Een 5 | - | - | - | - | + | + | 2.289 |
| 151 | Een 6 | - | + | - | - | + | + | 1.533 |
| 152 | Emai 11 | - | + | - | + | - | + | 1.087 |
| 153 | Emai 12 | - | - | - | + | + | + | 1.424 |
| 154 | Emai 18 | - | + | - | + | - | + | 1.672 |
| 155 | Emai 23 | - | + | + | + | - | + | 1.021 |
| 156 | Emai 27 | - | + | - | + | - | + | 0.872 |
| 157 | Emai 352 | - | + | - | + | - | + | 1.065 |
| 158 | Emai 580 | - | + | - | + | - | + | 1.398 |
| 159 | Emai 596 | - | + | - | + | + | + | 1.037 |
| 160 | Hua 2533 | - | + | + | + | - | + | 0.735 |
| 161 | Hua 2566 | - | - | + | + | - | + | 1.066 |
| 162 | Jingfumai 1 | - | + | - | + | - | + | 0.550 |
| 163 | Jingmai 103 | - | + | - | + | - | U | 1.292 |
| 164 | Jingzhou 66 | - | + | + | + | + | + | 1.890 |
| 165 | Wuhan 1 | - | - | - | + | - | + | 1.229 |
| 166 | Xiangmai 25 | - | - | - | + | - | + | 1.677 |
| 167 | Xiangmai 55 | - | - | - | + | - | + | 2.045 |
| 168 | CJ9306 | - | + | - | + | + | + | 1.324 |
| 169 | Ning 7840 | - | + | - | - | - | + | 1.976 |
| 170 | Ningmai 8 | - | + | - | + | - | + | 1.653 |
| 171 | Ningmai 9 | - | + | + | + | - | + | 0.841 |
| 172 | Ningmai 11 | - | - | + | - | + | + | 1.898 |
| 173 | Ningmai 13 | U | U | + | U | U | U | 0.653 |
| 174 | Ningmai 16 | - | - | - | + | + | + | 1.349 |
| 175 | Sumai 3 | - | + | - | - | + | + | 2.469 |
| 176 | Yang 05-117 | - | + | - | + | - | + | 1.125 |
| 177 | Yang 06-144 | - | + | + | + | - | + | 0.859 |
| 178 | Yang 06G86 | - | + | - | + | - | + | 1.132 |
| 179 | Yang 07-129 | - | U | - | + | U | - | 1.374 |
| 180 | Yang 07-15 | - | - | - | - | - | - | 1.925 |
| 181 | Yang 07-44 | - | + | - | + | - | + | 0.913 |
| 182 | Yang 07-49 | - | + | - | + | - | + | 0.793 |
| 183 | Yang 07-81 | - | + | - | + | - | + | 0.875 |
| 184 | Yangfumai 2 | - | + | - | + | - | + | 0.734 |
| 185 | Yangmai 11 | - | + | - | + | - | + | 0.589 |
| 186 | Yangmai 12 | - | + | - | + | - | + | 1.025 |
| 187 | Yangmai 13 | + | + | + | + | - | + | 0.719 |
| 188 | Yangmai 14 | - | + | - | + | - | + | 1.434 |
| 189 | Yangmai 15 | - | + | + | + | - | + | 0.971 |
| 190 | Yangmai 16 | - | - | - | + | - | + | 1.578 |
| 191 | Yangmai 17 | - | + | - | + | - | + | 0.833 |
| 192 | Yangmai 20 | - | + | - | + | - | - | 1.256 |
| 193 | Yangmai 22 | - | + | + | + | - | + | 0.641 |
| 194 | Yangmai 158 | - | + | - | + | - | + | 1.604 |
| 195 | Zhenmai 5 | - | + | + | + | - | + | 1.265 |
| 196 | Zhenmai 6 | - | + | - | + | + | + | 1.369 |
| 197 | Zhenmai 168 | - | + | - | + | - | + | 1.236 |
| 198 | Chuanmai 42 | - | + | + | + | - | + | 0.660 |
| 199 | Chuanmai 42 selection | - | + | + | + | - | + | 0.581 |
| 200 | Chuanmai 43 | - | + | + | + | - | + | 0.672 |
| 201 | Chuanmai 50 | - | + | + | + | - | + | 1.217 |
| 202 | Chuanmai 51 | - | + | + | + | - | + | 1.558 |
| 203 | Chuanmai 52 | U | + | + | + | - | + | 0.808 |
| 204 | Mianmai 37 | - | + | + | + | - | + | 0.770 |
| 205 | Mianmai 42 | - | - | + | + | - | + | 1.050 |
| 206 | Mianyang 99-3 | - | + | + | + | - | + | 1.081 |
| 207 | Mianyang 99-7 | - | + | + | + | - | + | 0.889 |
| 208 | Mianmai 185 | - | + | + | + | - | + | 0.887 |
| 209 | Mianmai 1403 | - | + | + | + | - | + | 0.794 |
| 210 | Neimai 8 | - | + | + | + | - | + | 0.913 |
| 211 | Shuangkang 7438 | - | + | + | + | - | + | 1.227 |
| 212 | Xikemai 2 | - | + | + | + | - | + | 0.807 |
| 213 | Xikemai 4 | - | + | + | + | - | + | 0.442 |
| 214 | Xikemai 5 | - | + | + | + | - | + | 0.803 |
| 215 | XK0106-1-0806 | - | + | + | + | - | - | 1.069 |
| 216 | Lantian 12 | - | + | + | - | - | + | 1.515 |
| 217 | Lantian 13 | - | + | + | - | - | + | 1.140 |
| 218 | Lantian 15 | - | + | + | - | + | + | 1.357 |
| 219 | Lantian 17 | - | + | + | + | - | + | 0.754 |
| 220 | Lantian 18 | - | + | - | - | U | + | 1.004 |
| 221 | Lantian 21 | - | + | + | - | - | + | 1.713 |
| 222 | Lantian 22 | - | + | + | + | - | + | 1.172 |
| 223 | Lantian 23 | - | + | + | + | - | + | 1.578 |
| 224 | Lantian 26 | - | + | + | - | - | + | 1.643 |
| 225 | Ningchun 4 | - | + | - | + | - | + | 1.200 |
| 226 | Ningchun 43 | - | - | + | + | - | + | 1.240 |
| 227 | Ningchun 47 | - | + | + | - | - | + | 1.542 |
| 228 | Ningdong 10 | - | - | - | + | - | + | 1.120 |
| 229 | Ningdong 11 | - | - | - | + | - | + | 1.580 |
| 230 | Gamenya | U | - | U | - | U | - | 2.510 |
| 231 | CROC_1/AE.SQUARROSA (205)//KAUZ/3/SASIA/4/TROST | - | - | + | + | - | + | 1.560 |
| 232 | MONARCA F2007/KRONSTAD F2004 | - | - | + | - | - | + | 1.440 |
| 233 | PBW343*2/KUKUNA//PBW343*2/KUKUNA/3/PBW343 | - | - | - | + | - | + | 0.949 |
| 234 | KS82W418/SPN//WBLL1/3/BERKUT | + | + | - | U | - | + | 0.854 |
| 235 | CNDO/R143//ENTE/MEXI75/3/AE.SQ/4/2*FCT/5/KAUZ*2/YACO//KAUZ/6/BERKUT | - | - | + | - | - | + | 1.357 |
| 236 | T.DICOCCON PI94625/AE.SQUARROSA (372)//TUI/CLMS/3/2*PASTOR/4/EXCALIBUR | - | + | + | + | - | + | 1.138 |
| 237 | NG8675/CBRD//MILAN/3/SAUAL/6/CNDO/R143//ENTE/MEXI_2/3/AEGILOPS SQUARROSA (TAUS)/4/WEAVER/5/2*PASTOR | - | - | - | U | - | + | 1.611 |
| 238 | Mayoor | + | - | + | + | - | + | 1.036 |
| 239 | Ocoroni | - | - | - | + | - | + | 0.770 |
| 240 | SYN1 | + | - | + | + | - | + | 0.606 |

“+”, resistance allele of the corresponding QTL; “-”, susceptibility allele of the corresponding QTL; “U”, uncertain.

Table S2 FSB severity of the nature population with different QTL-combinations.

| Number of QTL | Number of cultiv2ars (lines) | Mean of lesion length (cm)^#^ | Min | Max | SD | Std Error |
| --- | --- | --- | --- | --- | --- | --- |
| 0 | 1 | 1.93 | - | - | - | - |
| 1 | 4 | 1.28a | 0.98 | 1.75 | 0.35 | 0.17 |
| 2 | 38 | 1.19b | 0.60 | 2.31 | 0.45 | 0.07 |
| 3 | 90 | 1.11b | 0.35 | 2.47 | 0.41 | 0.04 |
| 4 | 89 | 0.89b | 0.37 | 1.769 | 0.29 | 0.03 |
| 5 | 5 | 0.72c | 0.57 | 0.89 | 0.114 | 0.05 |

^#^Values followed by different letters are significant by different in FSB severity among genotypes (P < 0.05).

Table S3 Differentially expressed genes infected by Fusarium spp. compared to those in the mock.

| Gene ID | Chr^a^ | Start | End | Length | Annotation^b^ | NIL-S Fp 3 days^c^ | NIL-S Fp 5 days | NIL-R Fp 3 days | NIL-R Fp 5 days | Chara Fp 36 hours |
| --- | --- | --- | --- | --- | --- | --- | --- | --- | --- | --- |
| TraesCS1D02G381800 | 1D | 457069625 | 457074809 | 2887 | Protein kinase-like domain | 3.97 | 4.08 | 2.16 | 22.28 | 2.61 |
| TraesCS1D02G382200 | 1D | 457246450 | 457247651 | 979 | Protein of unknown function DUF1639 | 0.58 | 0.81 | 1.87 | 6.78 | 1.73 |
| TraesCS1D02G382500 | 1D | 457539745 | 457541403 | 1452 | Protein of unknown function DUF1639 | 0.68 | 0.72 | 0.54 | 0.46 | 2.66 |
| TraesCS1D02G382600 | 1D | 457545967 | 457549313 | 2080 | TRAF-like | 1.00 | 0.15 | 1.00 | 0.64 | 2.59 |
| TraesCS1D02G383600 | 1D | 458132985 | 458134163 | 1179 | tRNA dimethylallyltransferase | 1.11 | 0.77 | 0.46 | 10.89 | 3.46 |
| TraesCS1D02G383900 | 1D | 458161019 | 458161261 | 243 | - | 1.00 | 1.00 | 1.00 | 24.98 | 0.00 |
| TraesCS1D02G384000 | 1D | 458167037 | 458167279 | 243 | - | 0.96 | 1.29 | 2.06 | 2.69 | 1.40 |
| TraesCS1D02G384400 | 1D | 458257201 | 458257812 | 612 | - | 2.81 | 0.87 | 0.36 | 1.47 | 0.90 |
| TraesCS1D02G384500 | 1D | 458280217 | 458280706 | 312 | - | 0.01 | 1.00 | 7.07 | 7.58 | 0.01 |
| TraesCS1D02G384600 | 1D | 458286036 | 458286266 | 231 | - | 0.99 | 1.84 | 0.61 | 4.81 | 0.71 |
| TraesCS1D02G384900 | 1D | 458481669 | 458483132 | 1464 | Nucleotide-diphospho-sugar transferases | 1.74 | 2.96 | 1.49 | 2.37 | 0.92 |
| TraesCS1D02G385100 | 1D | 458676188 | 458677503 | 849 | - | 2.34 | 0.11 | 1.51 | 0.59 | 0.56 |
| TraesCS1D02G385400 | 1D | 458784636 | 458785547 | 912 | Protein of unknown function DUF4228, plant | 0.82 | 2.42 | 1.02 | 1.06 | 1.20 |
| TraesCS1D02G386100 | 1D | 458878123 | 458882218 | 2476 | - | 1.08 | 0.21 | 2.70 | 1.17 | 1.00 |
| TraesCS1D02G386600 | 1D | 459519000 | 459523642 | 2187 | F-box domain | 8.80 | 0.25 | 0.34 | 0.51 | 1.95 |
| TraesCS1D02G387000 | 1D | 459831193 | 459838843 | 2720 | Oligopeptide transporter, OPT superfamily | 2.11 | 1.43 | 1.72 | 0.67 | 0.57 |
| TraesCS1D02G387500 | 1D | 460142745 | 460144820 | 1079 | FAS1 domain | 8.76 | 1.30 | 0.94 | 0.39 | 2.94 |
| TraesCS1D02G387700 | 1D | 460152897 | 460159561 | 363 | - | 6.18 | 1.37 | 1.00 | 2.56 | 8.75 |
| TraesCS1D02G388000 | 1D | 460190851 | 460193819 | 766 | Histone chaperone ASF1-like | 0.86 | 0.93 | 1.26 | 1.15 | 3.13 |
| TraesCS1D02G388800 | 1D | 460851253 | 460857684 | 3204 | NB-ARC | 0.98 | 2.21 | 0.49 | 5.24 | 1.29 |
| TraesCS3A02G168100 | 3A | 175237609 | 175240369 | 1673 | Domain of unknown function DUF1618 | 0.83 | 1.88 | 1.17 | 6.01 | 1.49 |
| TraesCS3A02G168400 | 3A | 175844695 | 175846642 | 1470 | Alpha/Beta hydrolase fold | 1.00 | 0.92 | 3.24 | 1.00 | 1.00 |
| TraesCS3A02G169000 | 3A | 176990481 | 176991053 | 573 | - | 1.06 | 9.20 | 0.03 | 2.94 | 4.13 |
| TraesCS3A02G169600 | 3A | 177702531 | 177704115 | 1585 | Legume lectin domain | 1.04 | 2.37 | 0.57 | 2.99 | 2.27 |
| TraesCS3A02G169800 | 3A | 177894086 | 177902506 | 3611 | P-loop containing nucleoside triphosphate hydrolase | 1.34 | 0.90 | 0.38 | 3.37 | 0.47 |
| TraesCS3A02G169900 | 3A | 178118523 | 178121422 | 919 | Dephospho-CoA kinase | 1.82 | 0.74 | 0.79 | 2.31 | 0.76 |
| TraesCS3A02G170300 | 3A | 178245198 | 178251375 | 2521 | C2 domain | 9.74 | 0.73 | 6.18 | 0.01 | 0.26 |
| TraesCS6B02G391800 | 6B | 666774485 | 666775270 | 786 | Transcription factor, MADS-box | 2.19 | 1.00 | 1.00 | 1.00 | 2.66 |
| TraesCS6B02G393500 | 6B | 668425716 | 668431387 | 2726 | MCM domain | 1.00 | 0.01 | 7.12 | 1.00 | 3.64 |
| TraesCS6B02G394300 | 6B | 669323182 | 669326524 | 1430 | - | 2.44 | 0.54 | 1.92 | 4.23 | 1.02 |
| TraesCS7A02G548100 | 7A | 722767794 | 722768819 | 1026 | Glycoside hydrolase, family 19, catalytic | 1.46 | 1.29 | 1.67 | 2.52 | 1.00 |
| TraesCS7A02G548900 | 7A | 723074814 | 723075326 | 513 | - | 1.00 | 1.00 | 1.00 | 1.00 | 4.94 |
| TraesCS7A02G549000 | 7A | 723255093 | 723258234 | 1339 | NAC domain | 1.66 | 2.10 | 1.75 | 9.18 | 0.50 |
| TraesCS7A02G549100 | 7A | 723259779 | 723265225 | 2580 | Glycoside hydrolase superfamily | 3.35 | 2.16 | 1.43 | 1.47 | 1.00 |
| TraesCS7A02G549300 | 7A | 723283231 | 723293633 | 2257 | Glycoside hydrolase superfamily | 3.00 | 1.16 | 1.63 | 2.45 | 1.02 |
| TraesCS7A02G549800 | 7A | 724082577 | 724090076 | 1980 | Catalase-like domain | 0.19 | 4.43 | 0.89 | 0.32 | 0.04 |
| TraesCS7A02G549900 | 7A | 724113049 | 724114569 | 873 | Catalase-like domain | 1.00 | 1.00 | 2.05 | 0.49 | 1.09 |
| TraesCS7A02G551900 | 7A | 725224414 | 725228601 | 3373 | NB-ARC | 2.25 | 1.11 | 0.85 | 1.68 | 1.27 |
| TraesCS7A02G552000 | 7A | 725230034 | 725234405 | 1957 | FAD/NAD(P)-binding domain | 1.43 | 1.23 | 0.98 | 2.00 | 6.07 |
| TraesCS7A02G552400 | 7A | 725433279 | 725435212 | 949 | High mobility group box domain | 0.91 | 0.82 | 1.02 | 0.89 | 13.89 |
| TraesCS7A02G552800 | 7A | 725612386 | 725613786 | 711 | BURP domain | 0.69 | 2.75 | 7.82 | 27.34 | 1.00 |
| TraesCSU02G035900 | Un | 30827997 | 30832807 | 4313 | Protein kinase-like domain | 6.14 | 2.17 | 0.13 | 0.63 | 0.81 |
| TraesCSU02G036400 | Un | 31094941 | 31096483 | 1543 | U box domain | 2.10 | 5.26 | 1.54 | 8.52 | 0.04 |
| TraesCSU02G037100 | Un | 31311249 | 31313722 | 1518 | F-box domain | 0.81 | 1.29 | 1.26 | 1.08 | 2.13 |
| TraesCSU02G037200 | Un | 31318914 | 31324234 | 3703 | RNA recognition motif domain | 2.05 | 0.44 | 0.31 | 0.74 | 2.14 |
| TraesCSU02G037600 | Un | 31480647 | 31484336 | 1816 | - | 0.13 | 1.00 | 4.22 | 0.76 | 1.00 |
| TraesCSU02G038200 | Un | 31625011 | 31626433 | 1350 | Transferase | 1.00 | 16.72 | 1.00 | 5.80 | 1.00 |
| TraesCSU02G038300 | Un | 31640297 | 31643228 | 2635 | Domain of unknown function DUF4220 | 0.40 | 1.00 | 3.68 | 2.78 | 0.97 |
| TraesCSU02G038700 | Un | 31723562 | 31725050 | 966 | Protein kinase-like domain | 1.00 | 6.77 | 1.00 | 1.00 | 1.00 |
| TraesCSU02G039100 | Un | 32038075 | 32038777 | 703 | Zinc finger C2H2-type | 0.52 | 7.71 | 2.41 | 0.96 | 1.00 |
| TraesCSU02G039200 | Un | 32042106 | 32042462 | 357 | Zinc finger C2H2-type | 1.00 | 3.44 | 0.03 | 1.00 | 1.27 |
| TraesCSU02G039600 | Un | 32132173 | 32132929 | 663 | Peptidase S8/S53 domain | 0.53 | 1.53 | 6.33 | 1.45 | 1.00 |
| TraesCSU02G039900 | Un | 32198478 | 32207377 | 5260 | Sin3, C-terminal | 14.33 | 8.86 | 22.78 | 0.66 | 0.05 |
| TraesCSU02G040200 | Un | 32287741 | 32290636 | 1914 | F-box domain | 0.07 | 3.23 | 0.02 | 1.00 | 1.00 |
| TraesCSU02G041500 | Un | 34103365 | 34130423 | 3210 | Leucine-rich repeat domain, L domain-like | 2.22 | 0.08 | 1.31 | 0.63 | 1.79 |
| TraesCSU02G041700 | Un | 34142009 | 34149000 | 3249 | - | 2.06 | 0.45 | 0.45 | 0.83 | 13.64 |
| TraesCSU02G041800 | Un | 34164758 | 34166407 | 1650 | Protein kinase-like domain | 1.81 | 2.09 | 1.84 | 4.82 | 0.34 |

^a^ Chr, Chromosome; ^b^ Gene annotations were referred to IWGSC RefSeq annotation v1.1; ^c^ S-NIL1-Fp 3 days, susceptible isolines infected by Fusarium spp. after 3 days; S-NIL1-Fp 5 days, susceptible isolines infected by Fusarium spp. after 5 days; R-NIL1-Fp 3 days, resistant isolines infected by Fusarium spp. after 3 days; R-NIL1-Fp 5 days, resistant isolines infected by Fusarium spp. after 3 days; Chara-Fp 36 hours, wheat culture ‘Chara’ infected by Fusarium spp. after 36 hours
